# Bacteria Isolated From Milk of Dairy Cows With and Without Clinical Mastitis in Different Regions of Australia and Their AMR Profiles

**DOI:** 10.3389/fvets.2021.743725

**Published:** 2021-11-04

**Authors:** Hulayyil Al-harbi, Shahab Ranjbar, Robert J. Moore, John I. Alawneh

**Affiliations:** ^1^Good Clinical Practice Research Group (GCPRG), School of Veterinary Science, The University of Queensland, Gatton, QLD, Australia; ^2^School of Veterinary Science, The University of Queensland, Gatton, QLD, Australia; ^3^School of Science, RMIT University, Bundoora, VIC, Australia; ^4^School of Veterinary Medicine, Murdoch University, Murdoch, WA, Australia

**Keywords:** mastitis, *Staphylococcus*, *Streptococcus*, *Enterobacteriaceae*, cattle, antimicrobial susceptibility, non-aureus staphylococci

## Abstract

Mastitis is the most common disease in dairy cattle worldwide. The objectives of this study were to estimate the prevalence of different bacterial species associated with mastitis from dairy herds located in geographically and climatically distinct zones in Australia, and to evaluate the antimicrobial susceptibility of the isolated bacteria. Quarter-level milk samples (*n* = 419) were collected from 151 mastitis cases and 268 healthy controls originating from 18 dairy herds located in tropical (Northern Queensland), subtropical (Southeast Queensland) and temperate zones (Victoria) between March and June 2019. Milk samples were cultured, and the isolated bacteria were grouped into six groups: *Enterobacteriaceae* spp.; *Streptococcus* spp.; *Staphylococcus aureus*, non-aureus staphylococci (NAS); *Bacillus* spp.; and Others. Mixed effects conditional logistic regression models were applied to quantify the association between the prevalence of each bacterial group and the herd zone and bulk milk tank somatic cell counts (BMTSCC). Of the 205 isolates, 102 (50%) originated from mastitis cases, and 103 (50%) from controls. Staphylococci were the most prevalent (NAS 32% and *S. aureus* 11%). Contagious mastitis bacteria were more prevalent in Victoria compared to Queensland dairy herds. NAS species (*P* < 0.001) were less prevalent in herds with BMTSCC >300,000 cells/mL compared with herds with low BMTSCC ≤150,000 cells/mL. *Enterobacteriaceae* and *Streptococcus* spp. groups showed high resistance rates to 1 (51 and 47%, respectively), and 2 (11 and 23%, respectively), antimicrobials. More than one third of the *Enterobacteriaceae* (48%) and Others (43%) groups spp. were resistant to at least three antimicrobials. This study provided a unique opportunity to investigate the prevalence of mastitis-associated bacteria in clinical cases and in apparently healthy controls. The findings of this study help inform mastitis control and antimicrobial stewardship programs aimed to reduce the prevalence of mastitis and antimicrobial resistance in dairy herds.

## Introduction

Bacterial bovine mastitis is the most common disease in the dairy cattle industry worldwide, causing significant welfare and economic implications (1). In Australia, the estimated total cost associated with each case of mastitis ranges between AU$47 and $427 per infected cow per year. This cost includes treatment, reduced milk production, reduced conception rates, and culling (2). Bacterial mastitis also impacts milk composition and quality, which adds to the economic burden (3).

The major mastitis bacteria in most countries are *Staphylococcus aureus, Streptococcus agalactiae, Streptococcus dysgalactiae, Streptococcus uberis*, and *Escherichia coli* (4). However, bacteria differ in relative prevalence between regions and countries (5). Therefore, systematic identification of the prevalent bacteria is recommended for successful mastitis control programs (6).

Individual cow and bulk milk tank (BMT) somatic cell counts (SCC) are commonly used to monitor changes in milk quality and mastitis progression in a herd (7, 8). A high individual cow SCC, with or without clinical signs, is an indicator of intramammary infection (IMI), while an elevated BMTSCC indicates an increased risk of mastitis in a herd (3). However, average SCC varies for different mastitis-associated bacteria. For example, *S. uberis* and *S. agalactiae* IMIs have lower SCCs compared to *S. aureus* IMI (9).

Bovine mastitis prevention and control programs are based on targeted antimicrobial treatments (10). However, antimicrobial treatment failure is of concern due extensive empirical use of antimicrobials and the increased prevalence of antimicrobial resistance (AMR) in mastitis pathogens (11). Recent epidemiological studies in Denmark and China have reported data on AMR profiles in bovine mastitis bacteria, and the use of antimicrobial susceptibility testing is increasingly recommended to guide the selection of the most appropriate treatment approach (1, 11). Consequently, monitoring of both the mastitis bacteria and their AMR profiles should be regularly undertaken at the local level to enable timely adjustment of the prevention and control strategies (12).

There is paucity of up-to-date data on AMR profiles in bovine mastitis bacteria in Australian dairy farms. Some bacteria are of public health concern, such as *S. aureus*, non-aureus staphylococci (NAS), *E. coli*, and streptococci, because these bacteria can be reservoirs for AMR genes, which can spread among other pathogenic and commensal bacteria in the dairy farm environment (13). To the authors' knowledge, there is a lack of data on potential regional differences in the prevalence of different mastitis bacterial species in the country. Such information is crucial to the successful management of mastitis strategies at the regional level. Previous surveys of mastitis-causing bacteria in lactating cows in Australia have been limited in geographical coverage (14–16). Therefore, the present study was conducted to estimate the prevalence of bacterial species isolated from mastitis and healthy animals from study herds located in geographically and climatically distinct zones in Australia and to attempt to quantify the association between the herd's BMTSCC and the prevalence of mastitis-associated bacteria. A further objective was to evaluate the antimicrobial susceptibility of the isolated bacteria from these locations.

## Materials and Methods

### Design and Settings

**Figure 1:**
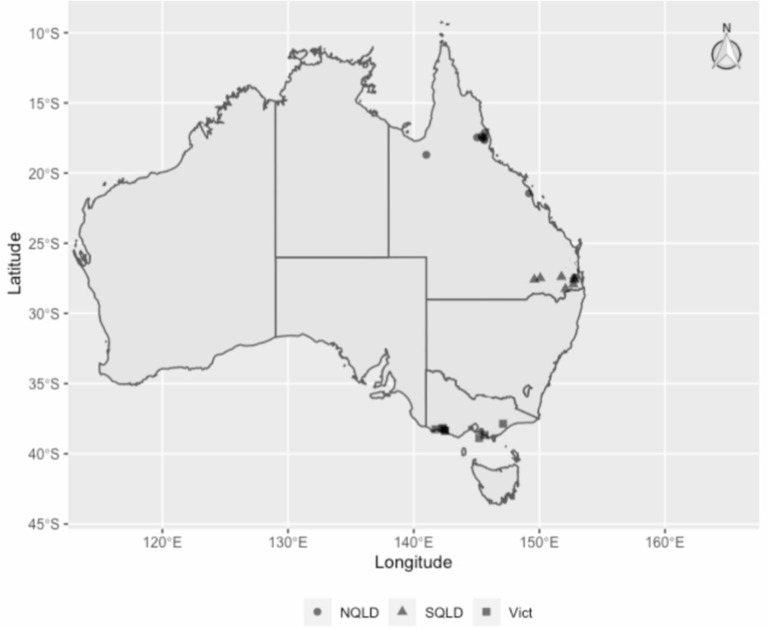
The study herds' regions and locations: circles for Northern Queensland (NQLD), triangles for Southeast Queensland (SQLD) and squares for Victoria (Vict).

This was a matched case-control study conducted between March and June 2019, involving 18 dairy herds (Figure 1) from three different climate zones in Australia as defined by the Australian Bureau of Meteorology (17): tropical, Northern Queensland (NQLD, *n* = 8 farms); subtropical, Southeast Queensland (SQLD, *n* = 5); and temperate, Victoria (*n* = 5). Within each zone, herd eligibility to be enrolled in this study was based on farm management willingness to participate, convenience in terms of logistics, and herd BMTSCC. In 2019, there were a total of 5,055 registered dairy herds in Australia. Of these, 3,462 (69%) were in Victoria and 7% in Queensland (18). Further information on national dairy herd size and milk production can be found on the Dairy Australia website (18). The study was conducted in accordance with the University of Queensland Animal Ethics and National Guidelines (animal ethics approvals: SVS/ANRFA/540/18 and SVS/043/18/TERRAGEN).

### Herd and Animal Enrolment

A 2-week rolling average of herd BMTSCC was used to enroll herds from each farm. According to the two-week average BMTSCC, herds were classified into three categories: ≤150 × 1,000 cells/mL; >150–300 × 1,000 cells/mL; and >300 × 1,000 cells/mL. These categories were used as an *a priori* assumption to represent different levels of animal exposure to potential pathogens circulating in these herds.

All milking cows were screened for clinical mastitis by the first and last author during the morning or afternoon milking. Prior to cups on, all quarters were stripped and checked for changes in milk appearance, texture and odor. The udder was also checked for tenderness, swelling and changes in color. From each herd, we aimed to identify and sample clinical mastitis cases and, where possible, match those with a comparable number of apparently healthy cows (controls). Mastitis cases were identified by clinical examination of cows, and were defined as the first occurrence of a mastitis event during the current lactation or a mastitis event occurring 21 days after a previous mastitis event that had clinically resolved or achieved clinical cure (19). A mastitis case was not a subclinical or chronic mastitis case. Healthy controls were defined as a lactating dairy cow at any stage of lactation that had not experienced a clinical or subclinical mastitis event and had not received antimicrobial treatment for any other disease in the 14 days prior to enrolment in the study. Mastitis and control cows were matched on age (+/− 2 years), days in milk (DIM; 0–100 DIM; 101–200 DIM; >201 DIM) and sampled quarter.

### Milk Sample Collection

Once the cases were identified, milk samples were collected (by first and last authors) from mastitic quarters and, where possible, an internal control milk sample. Internal control milk samples were from the same cow, predominantly from the contralateral, apparently healthy quarter that tested negative (i.e., zero score, below 200,000 SCC/mL) on the California Rapid Mastitis Test (20). The external control quarter was defined as for the internal control quarter but from the matched healthy control cow. Thus, for every mastitic milk sample, our aim was to collect two control samples: an internal (from a healthy quarter in the same cow), and an external (from the matched healthy cow).

All milk samples were collected aseptically. Teats were thoroughly washed and dried with single-use paper towel, and teat ends were scrubbed for several seconds with paper towel soaked in 70% ethanol. Two to three foremilk streams were stripped, then ~30 mL of milk was collected into a sterile tube that was immediately capped to avoid any contamination. Three aseptically collected samples were obtained from the same quarter: one aliquot was for the bacterial culture; the second aliquot was for the DNA extraction; and the third aliquot was for backup storage. The milk samples were immediately placed into a −20°C freezer until delivered to the University of Queensland Veterinary Laboratory Services (UQVLS) for culture and further investigations.

### Bacterial Culture

Frozen milk samples intended for microbiology investigation were thawed at room temperature. For bacterial isolation, 100 μL of milk were streaked onto sheep blood Columbia agar (SBA, P2133 Sheep Blood Columbia Agar Plates, Thermo Fisher, Brisbane, Australia) and MacConkey agar (MCA, PP2130 MacConkey No. 3 Agar Plates, Thermofisher, Brisbane, Australia), which were then incubated aerobically at 37°C for up to 48 h. Samples with up to two colony types, regardless of the number of colonies, were considered positive cultures and the predominate colony type was selected for subsequent analyses. Samples with more than two colony types were considered contaminated and were discarded (*n* = 0). The isolated bacteria were sub-cultured onto SBA and MCA plates and incubated aerobically at 37°C for another 18–24 h. The pure isolates were identified using phenotypic tests, including Gram stain, oxidase, indole, and catalase tests. The isolates were then transported at room temperature to the Department of Agriculture and Fisheries, Biosecurity Queensland laboratory for further identification using matrix-assisted laser desorption ionization-time of flight mass spectrometry (MALDI-TOF MS).

For MALDI-TOF MS analysis, samples were prepared using cells from a single colony of fresh overnight SBA culture and were prepared according to the standard protocol provided by the manufacturer (Bruker Daltonik, Bremen, Germany). Briefly, using a toothpick, a small amount of biological material from a single colony from the plates was smeared as a thin film directly onto one spot of the MALDI steel target plate. Using the same toothpick, a replicate second spot was made on the target plate. The biological material was overlaid with 1 μL of matrix solution (a-cyano-4-hydroxy-cinnamic acid diluted in 50% acetonitrile and 2.5% trifluoracetic acid) within 1 h and allowed to dry at room temperature. The target plate was then loaded on a Bruker MALDI-TOF Biotyper and identified by comparing the mass spectral protein detection pattern with the reference patterns in the database. A MALDI-TOF MS score of >2.0 was accepted as representing a reliable identification (21).

### DNA Extraction From Milk Samples

After thawing the milk sample at room temperature, genomic DNA was extracted using the DNeasy PowerFood Microbial Kit (QIAGEN Chadstone, Victoria, Australia), with minor modifications. An 8 mL milk sample was transferred into a 10 mL centrifuge tube (Thomas scientific, Swedsboro, New Jersey, USA), which was then centrifuged for 15 min at 4°C at 20,000 x g. The cream layer and supernatant were discarded, avoiding the pellets. The remaining pellets were resuspended in 1 mL of sterile 0.85% NaCl solution and transferred into a 2 mL sterile Eppendorf tube (Eppendorf Quality ^TM^, Germany), which was then centrifuged for 10 min at 4°C at 14,000 x g. The supernatant was discarded, and the remaining cell pellets were resuspended in a 450 μL lysis buffer and incubated for 10 min at 65°C. Proteinase K (QIAGEN Chadstone, Victoria, Australia) (25 μL) was added and then the tube was incubated for 20 min at 65°C. This treatment increased the final DNA quantity compared to the untreated samples (data not shown). Thereafter, the entire component was transferred to the Powerbead tube and secured horizontally to a vortex adapter (Vortex-2 Genie®) and vortexed at a maximum speed for 10 min. After vortex and centrifuge steps to remove protein and other inhibitors, purified DNA was then eluted using 100 μL of elusion buffer and the concentration and purity of isolated genomic DNA was evaluated based on optical densities at 230, 260, and 280 nm wavelength using a NanoDrop ND-1000 spectrophotometer (Thermo Scientific NanoDrop ^TM^).

### PCR for *Mycoplasma bovis*

The PCRs were performed at the Elizabeth Macarthur Agricultural Institute, NSW. The DNA extracts were tested for the presence of *Mycoplasma bovis* as pools of 12, except for samples 140, 141, and 143 that were tested as a pool of three. If *M. bovis* was detected in a pool, each sample of the positive pool was tested individually. PCR reactions were performed in a 20 μL volume and contained 1 μM MbovF and MbovR primers and 0.25 μM probe as previously described (22), 1 × Environmental mastermix (ThermoFisher Scientific) and molecular grade water. Thermal cycling was carried out on a QuantStudio S5 thermal cycler (ThermoFisher Scientific) and cycling parameters consisted of 1 × cycle at 95°C for 10 min, followed by 45 × cycles of 95°C for 15 s and 60°C for 60 s. Plasmid standards and negative controls were included in each run.

Plasmid standards were created by cloning the *M. bovis* PCR product into TOPO TA vector (ThermoFisher Scientific). Briefly, a portion of the *uvr*C gene was amplified using the MbovF and MbovR primers described above, but in a conventional PCR format using BioTaq polymerase (Bioline). The amplified product was purified using the Qiaquick DNA purification kit (Qiagen) and ligated into the TOPO TA cloning vector according to the manufacturer's instructions. The ligated plasmid was transformed into *E. coli* TOP10 cells and transformants were screened for the presence of inserts. Plasmid DNA was extracted from positive transformants using the Qiaprep Spin Miniprep kit (Qiagen) and the construct verified via Sanger sequencing at the Australian Genomic Research Facility (AGRF, Sydney). Plasmid standards consisted of serial 10-fold dilutions ranging from 200 fg/μL to 0.02 fg/μL.

The limit of detection (LOD) of the *M. bovis* qPCR assay was determined using 8 replicates of each of the plasmid standards. The LOD was defined as the point at which 95% of the replicates were positive and equated to 208 gene copies per μL of sample.

### Antimicrobial Susceptibility Test

The antibiotic susceptibility of isolates was determined using the disc diffusion method, using Muller Hinton media according to the guidelines of the Clinical and Laboratory Standards Institute (CLSI) (23). Fastidious organisms such as *Streptococcus* spp. require Muller Hinton medium supplemented with additional nutrients (5% sheep blood). Inhibition zones were measured using a ruler, and the interpretation of zone diameter was carried out according to the CLSI guidelines (24, 25).

### Antimicrobial Discs

The discs containing antimicrobial compounds were from Oxoid^TM^ (Thermofisher Brisbane, Australia). The panels of antimicrobials tested were the current panels that are routinely used in the UQVLS, and the current minimal inhibitory concentration recommended by CLSI guidelines (24, 25). The antimicrobials used for both Gram-positive and Gram-negative bacteria were: tetracycline (30 μg); cotrimoxazole (25 μg); enrofloxacin (5 μg); gentamicin (10 μg); amoxicillin-clavulanic acid (30 μg); cephalothin (30 μg); pen-novobiocin (40 μg); and ceftiofur (30 μg). The antimicrobials used for Gram-positive bacteria were penicillin (10 μg), erythromycin (15 μg), and oxacillin (1 μg); and for Gram-negative, ampicillin (10 μg), neomycin (30 μg), and chloramphenicol (30 μg). Gentamicin, enrofloxacin, and chloramphenicol were also selected, based on their importance to public health. *E. coli* American Type Culture Collection (ATCC) 25922 and *S. aureus* ATCC 25923 were used as reference strains for quality assurance.

### Data Management

Prior to data analyses, the results of the bacteriological analyses of all quarter milk samples (i.e., udder quarters) were divided into groups (**Table 2**): *Enterobacteriaceae* spp. group; *Streptococcus* spp. group; *S. aureus* group, NAS group; *Bacillus* spp. group; and Others group. For the antimicrobial susceptibility test, *Corynebacterium* spp. were excluded due to their long incubation period. Samples with no microbial growth were categorized as no growth (NG), while unidentifiable (using MALDI-TOF) isolates were categorized into NO ID group. The final dataset comprised of the following explanatory variables: (a) quarter status (categorical variable with two levels, case or control); (b) herd's region/ climate zone (a categorical variable with three levels: NQLD [tropical], SQLD [subtropical] and Victoria [temperate]); (c) herd's BMTSCC (a categorical variable with three levels: ≤ 150,000 cells/mL; 150,000–300,000 cells/mL, and >300,000 cells/mL); (d) match indicator (a continuous variable common for matched cases and controls); (e) animal identification number; (f) cultured isolate name (if applicable); (g) isolate antimicrobial sensitivity profile; and h) identified bacterial group (a categorical variable as defined above).

### Statistical Analysis

The distribution of collected milk samples from each region/zone (referred to as region hereafter) as a function of BMTSCC categories and milk sample type were summarized as counts and percentages of positive culture “milk samples which had bacterial growth on agar plate.” The homogeneity of sample counts and proportions of positive cultures across regions and milk sample type were compared using *Chi-*squared test for independence or Fisher's exact test implemented in R (26). Mixed effects conditional logistic regression models were used to quantify the association between the prevalence of each bacterial group and herd location after adjusting for herd average BMTSCC and, thus, no intercepts were estimated. Considering the study design we have used, a conditional logistic regression was an appropriate modeling approach (27). The mixed effects logistic regression models were fitted using the coxme package (28) implemented in R. Cow nested within match indicator was fitted as random effect in all models. The coxme function fits the following model:

(1) $$\begin{array}{r} \lambda_{\text{i}}\left(\text{t}\right)=\;{\;\lambda }_{0}\left(t\right){\text{e}}^{{\text{X}}_{\text{i}}\beta +{\text{Z}}_{\text{j}}\text{b}} \end{array}$$

where λ_0_ is an unspecified baseline hazard function at time *t* (time was constant at 1 to fit the conditional logistic regression model); *X*_*i*_ and *Z*_*j*_ are the design matrix for β as vector of fixed effects coefficients for quarter status, herd's region and herd's BMTSCC; and *b* as a vector of random effects coefficient for cow nested within match indicator. The random effects distribution was modeled as a log-normal distribution with a mean of zero and a variance of σ^2^. The random effects distribution was based on Akaike Information Criterion (AIC) and model log-likelihood statistics. For AMR analyses, *Chi-*squared and Fisher's exact tests were performed to identify significance in the proportion of AMR patterns between bacterial groups and the isolates within the groups. All bacterial species that were represented by less than two isolates in this study were excluded, and two-side p-values obtained by Monte-Carlo simulation (*n* = 2,000) of at least 0.05 were considered to be significant. Statistical analysis was conducted using stats package implemented in R. To visualize the overall multidrug resistant (MDR) patterns (co-occurrences) between bacterial species and antimicrobial agents, two matrices were constructed: one bimodal, and another square. In the first matrix, bacterial groups formed rows (in social network analysis (SNA) terminology farms would be called “actors” and antimicrobial agents would be called “affiliates”). The presence of AMR to a given antimicrobial agent and an isolate formed the “ties,” and the count of isolates resistant to a given antimicrobial agent formed the “weights” in the matrix. Ties were considered as undirected and were dichotomised; 1 indicating the presence of the tie and 0 otherwise. The eigenvector centrality score for each isolate and antimicrobial was estimated and used to identify actors and affiliates that are connected to many other actors or affiliates, which are, in turn, connected to many others (29). Therefore, the eigenvector score in this context is a measure of an actor's or an affiliate's importance in the network (29). The network was visualized using Fruchterman-Reingold layout. For the second matrix, antimicrobial agents formed the rows and the columns. The co-resistance to antimicrobial agents for a given isolate that belongs to a bacterial group formed the ties, and the count of isolates co-resistant to a given antimicrobial agent formed the weights in the matrix. Ties were considered as undirected and were dichotomised; 1 indicating the presence of the tie and 0 otherwise. Multidimensional scaling and shortest path matrix as the distances between nodes for matrix one and Pearson's correlation coefficient and the “leading eigenvector” method (30) or matrix two were used for generating the coordinates and identify AMR pattern “communities” in the networks. Social network analysis and visualization was carried out using the igraph package (31) implemented in R.

## Results

A total of 18 herds were investigated: 8 from NQLD, 5 from SQLD, and 5 from Victoria. All herds calved year-round and milked, on average [median], 370 (range = 275–750 cows), mixed-age Holstein-Friesian (HF) and HF-crossed dairy cattle. Mean cow age (Standard deviation [SD]) and DIM (SD) were 4.5 years (1.6) & 96 days (46), 4.5 years (1.4) & 78 days (28), and 4.1 years (1.2) & 76 days (32) for NQLD, SQLD, and Victoria, respectively. NQLD herds were predominantly pasture-fed while SQLD were fed pasture and partial mixed ration (PMR) on feed pad. Victorian herds grazed pasture supplemented with grain feeding in bail. From the study herds, 151 clinically mastitic quarters were sampled, and an additional 107 and 161 internal and external controls, respectively, for a total 419 milk samples (Table 1).

**Table 1:** Distribution and percentage of milk samples and positive cultures for each sample type stratified by herd's region and herd's bulk milk tank somatic cell counts category (BMTSCC).

**Herd region (zone)**	**Herd BMTSCC (x 1,000/mL)**	**n Herds**	**Milk samples**								***p*-value^a^**	***p*-value^b^**
			**Overall**		**Mastitis**		**Int. C**		**Ext. C**			
			**Total (n)**	Positive; n (%)^*^	**Total (n)**	Positive; n (%)^*^	**Total (n)**	Positive; n (%)^*^	**Total (n)**	Positive; n (%)^*^		
NQLD (tropical)	≤150	2	35 (25%)	16 (11%)	12	4 (3%)	8	3 (2%)	15	9 (6%)		
	150–300	4	73 (51%)	47 (33%)	26	21 (15%)	22	12 (8%)	25	14 (10%)		
	>300	2	34 (24%)	20 (14%)	9	5 (4%)	9	5 (4%)	16	10 (7%)	0.702	
	Subtotal	8	142 (100%)	83 (58%)	47	30 (64%)	39	20 (51%)	56	33 (59%)		0.499
SQLD (subtropical)	≤150	2	62 (44%)	33 (24%)	25	17 (12%	10	4 (3%)	27	12 (9%)		
	150–300	2	65 (46%)	35 (25%)	22	19 (14%)	18	8 (6%)	25	8 (6%)		
	>300	1	13 (9%)	8 (6%)	4	3 (2%)	5	4 (3%)	4	1 (1%)	0.386	
	Subtotal	5	140 (100%)	76 (54%)	51	39 (76%)	33	16 (48%)	56	21 (38%)		<0.001
Victoria (temperate)	≤150	2	42 (31%)	13 (10%)	15	8 (6%)	12	3 (2%)	15	2 (1%)		
	150–300	2	74 (54%)	50 (36%)	32	26 (19%)	16	9 (7%)	26	15 (11%)		
	>300	1	21 (15%)	7 (5%)	6	6 (4%)	7	1 (1%)	8	0 (0%)	0.702	
	Subtotal	5	137 (100%)	70 (51%)	53	40 (75%)	35	13 (37%)	49	17 (35%)		<0.001
Total^£^			419 (100%)	229 (55%)	151 (36%)	109 (72%)	107 (26%)	49 (46%)	161 (38%)	71 (44%)		<0.001

*Milk samples were collected from 151 clinical mastitis quarters and 268 apparently healthy control quarters from 18 dairy herds located in Northern Queensland (NQLD), Southeast Queensland (SQLD) and Victoria between March and June 2019*.

*Key: BMTSCC, Bulk milk tank somatic cell count; n, number; Int. C, Internal controls; Ext. C, External controls*.

a *Chi square comparison of the distribution of sample types (mastitis, internal and external controls) by herd's bulk somatic cell count in the given herd region*.

b *Chi square comparison of positive culture rates across the sample types within each region and in the total population*.

* *Percentage of subtotal*.

£ *Percentage of total (n)*.

**Figure 2:**
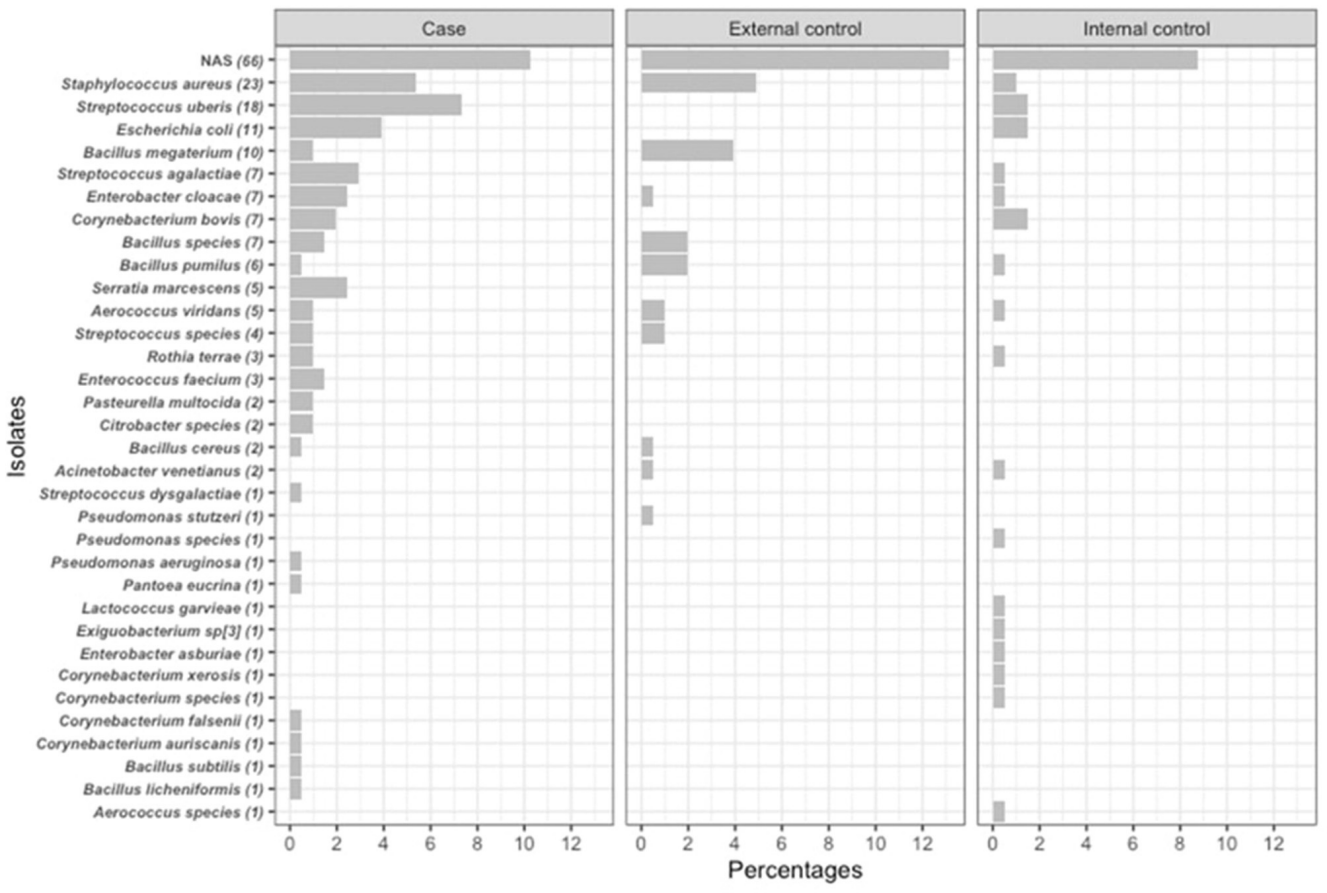
Counts and percentages of isolates (*n* = 205) cultured from quarter-level milk samples (*n* = 419) obtained from mastitis cases (*n* = 102), external (*n* = 61) and internal (*n* = 42) controls. Milk samples were obtained from dairy cattle originating from 18 dairy herds in Northern Queensland, Southeast Queensland and Victoria between March and June 2019.

The distribution of milk sample type (mastitis, internal or external controls) and percentage of positive cultures stratified by herd's region and BMTSCC category are summarized in Table 1. A total of 419 milk samples were collected from the overall study population. Milk sample types were evenly distributed across the BMTSCC categories in the three regions (*P* > 0.05). Of all 419 samples, 229 (55%) were culture positive without any significant differences across regions. The rate of positive cultures was higher in mastitis samples in the total population compared to internal and external controls, respectively (72 vs. 46% and 44%, *P* < 0.001), as well as in SQLD (76 vs. 48% and 38%, *P* < 0.001) and Victoria (75 vs. 37% and 35%, *P* < 0.001); however, this difference was not statistically significant in NQLD samples (64 vs. 51% and 59%, *P* = 0.50). Of the 229 positive cultures, 205 isolates (90%) were identified using MALDI-TOF and, of these, 50% (102/205) of isolates were from mastitis cases. The most common bacteria isolated from mastitis cases were NAS (10%), *S. uberis* (7%)*, S. aureus* (5%), and *E. coli* (4%; Figure 2). The most common bacteria isolated from control samples were NAS (22%) and *S. aureus* (5%). The prevalence of bacterial species was different between regions (*P* < 0.001). In NQLD, the most common bacterial species were NAS (42%), *S. uberis* (8%), and *E. coli* (6%). In SQLD, the most common bacterial species were NAS (37%), *S. uberis* (11%), and *Corynebacterium bovis* (7%). In Victoria, the most common bacterial species were *S. aureus* (30%), *S. agalactiae* (13%), NAS (13%), *E. coli* (9%), and *S. uberis* (7%) (Table 2 and Figure 2). *S. chromogenes* was the most common NAS species detected in mastitis and control samples (Supplementary Table 1). *Mycoplasma bovis* DNA was not detected within any of the pooled milk samples.

**Table 2:** Distribution of the identified bacterial species by bacterial group and region.

**Bacterial group**	**Northern Queensland**	**Southeast Queensland**	**Victoria**	**Total (%)^§^**
The total number of identified isolates (% of total)	78 (38%)	73 (36%)	54 (26%)	205 (100%)
*Enterobacteriaceae*; n (% of total)	12 (15%)	8 (11%)	7 (13%)	27 (13%)
*Escherichia coli*	5	1	5	11
*Enterobacter cloacae*	0	7	0	7
*Serratia marcescens*	5	0	0	5
*Enterobacter asburiae*	1	0	0	1
*Citrobacter species*	0	0	2	2
*Pantoea eucrina*	1	0	0	1
*Staphylococcus aureus*; n (% of total)	2 (3%)	5 (7%)	16 (30%)	23 (11%)
*Streptococcus* spp.; n (% of total)	10 (13%)	8 (11%)	12 (22%)	30 (15%)
*Streptococcus uberis*	6	8	4	18
*Streptococcus agalactiae*	0	0	7	7
*Streptococcus dysgalactiae*	1	0	0	1
*Streptococcus* species	3	0	1	4
Non-aureus staphylococci; n (% of total)	32 (42%)	27 (37%)	7 (13%)	66 (32%)
*Staphylococcus chromogenes*	8	22	0	40
*Staphylococcus haemolyticus*	12	1	3	16
*Staphylococcus warneri*	0	0	2	2
*Staphylococcus sciuri*	1	0	1	2
*Staphylococcus simulans*	0	2	0	2
*Staphylococcus equorum*	1	0	0	1
*Staphylococcus hominis*	0	1	0	1
*Staphylococcus hyicus*	0	1	0	1
*Staphylococcus xylosus*	0	0	1	1
*Bacillus* spp.; n (% of total)	15 (19%)	7 (10%)	5 (9%)	27 (13%)
*Bacillus megaterium*	4	6	0	10
*Bacillus pumilus*	5	0	1	6
*Bacillus cereus*	0	1	1	2
*Bacillus licheniformis*	0	0	1	1
*Bacillus subtilis*	0	0	1	1
*Bacillus* species	6	0	1	7
Others; n (% of total)	7 (9%)	18 (25%)	7 (13%)	32 (16%)
*Acinetobacter venetianus*	0	2	0	2
*Aerococcus* species	1	0	0	1
*Aerococcus viridans*	0	3	2	5
*Corynebacterium auriscanis*	0	1	0	1
*Corynebacterium bovis*	0	5	2	7
*Corynebacterium falsenii*	1	0	0	1
*Corynebacterium* species	0	0	1	1
*Corynebacterium xerosis*	0	1	0	1
*Enterococcus faecium*	0	3	0	3
*Exiguobacterium* sp[3]	0	1	0	1
*Lactococcus garvieae*	1	0	0	1
*Pasteurella multocida*	1	0	1	2
*Pseudomonas aeruginosa*	0	1	0	1
*Pseudomonas species*	1	0	0	1
*Pseudomonas stutzeri*	0	1	0	1
*Rothia terrae*	2	0	1	3

*Bacterial species were cultured from milk samples collected from 151 clinical mastitis quarters and 268 apparently healthy control quarters from 18 dairy herds located in Northern Queensland, Southeast Queensland, and Victoria between March and June 2019*.

§ *Percentages were calculated out of the column total*.

**Figure 3:**
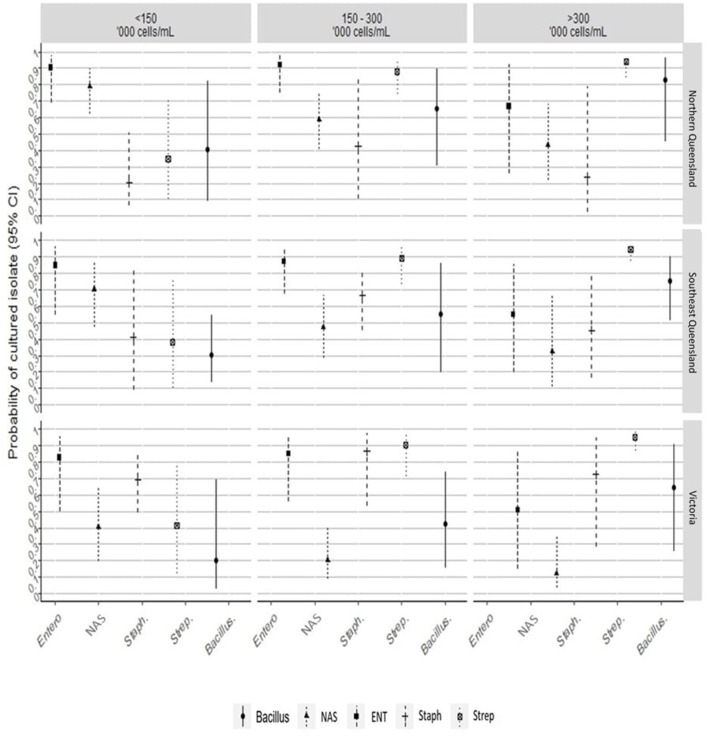
The line plot shows the probability of culturing a bacterial group from mastitis and healthy quarters, between herd's region, and average bulk milk somatic cell counts. Entero/ENT, *Enterobacteriaceae*; NAS, non-aureus staphylococci; Staph, *Staphylococcus aureus*; Strep, *Streptococcus* spp.

The results of the multivariate mixed effects conditional logistic regression models, quantifying the effects of herd's region and BMTSCC on the odds and the probability of isolating a bacterial group from mastitis quarters compared to control quarters, are presented in Figure 3 and Supplementary Tables 2, 3. After adjusting for the effect of herd's region and herd's BMTSCC, the odds of culturing *Enterobacteriaceae* spp. group from quarters with mastitis increased by 6.7-fold (Odd ratio [OR] = 6.7, 95% CI = 2.70, 16.72; *P* < 0.001), compared with control quarters. This translates into an adjusted probability of 87% (95% CI = 73%, 94%). Compared to herds from NQLD, the probability of culturing *Enterobacteriaceae* spp. group from mastitis quarters from herds in Victoria (33%, 95% CI = 16%, 56%) and SQLD (38%, 95% CI = 19%, 60%) did not differ (Supplementary Table 3). Milk samples from mastitis quarters from herds with an average BMTSCC of 150–300,000 cells/mL (54%, 95% CI = 34%, 73%) had the highest probability of culturing *Enterobacteriaceae* spp. compared with samples from herds with BMTSCC of >300,000 cells/mL (17%, 95% CI = 2%, 63%).

For the NAS group, the odds of culturing NAS species from mastitis quarters were ~50% (OR = 0.49, 95% CI = 0.24, 0.99; *P* < 0.05; adjusted probability 33%, 95% CI = 19%, 50%) lower than from control quarters. The odds of culturing NAS were influenced by region and BMTSCC. The probability of culturing NAS group from mastitis milk samples collected from Victorian herds (13%, 95% CI = 4%, 34%; *P* < 0.001) was lower than from SQLD (39%, 95% CI = 18%, 65%; *P* < 0.001). Similarly, milk samples from herds with BMTSCC of 150–300,000 cells/mL (20%, 95% CI = 8%, 41%; *P* < 0.001) or >300,000 cells/mL (14%, 95% CI = 4%, 38%; *P* < 0.001) had a lower probability of culturing NAS from mastitis cases compared with controls (Figure 3).

The odds of culturing *S. aureus* from quarters with mastitis was higher by at least 2-fold (OR = 2.08, 95% CI = 0.59, 7.34; *P* = 0.26) compared with control quarters. The odds of isolating *S. aureus* from Victoria (OR = 6.6, 95% CI = 1.00, 57.08) was high (*P* < 0.05; Figure 3 and Supplementary Table 2). The odds of culturing *Streptococcus* spp. from quarters with mastitis was 9.37-fold (OR = 9.37, 95% CI = 3.54, 24.85; *P* < 0.001) higher compared with controls. This translates into an adjusted probability of 90% (95% CI = 78%, 96%). The probability of culturing *Streptococcus* spp. from mastitis milk was also noticeably influenced by BMTSCC. The probability of culturing *Streptococcus* spp. from herds with BMTSCC of 150,000–300,000 cells/mL (93%, 95% CI = 60%, 99%) or >300,000 cells/mL (97%, 95% CI 76%, 100%) was higher (*P* < 0.05) compared with herds with BMTSCC of ≤150,000 cells/mL (Figure 3). For *Bacillus* spp. group, the odds of culturing from quarters with mastitis was at least three times more likely (OR = 3.62, 95% CI = 1.05, 12.45; *P* = 0.04), but neither herd's region nor herd's BMTSCC influenced the probability of culturing this group compared with controls (Supplementary Table 3). The odds of the No growth group in mastitis quarters were ~50% (OR = 0.43, 95% CI = 0.29, 0.64; *P* < 0.001; adjusted probability 30%, 95% CI = 22%, 39%) lower than in control quarters, but neither herd's region nor herd's BMTSCC influenced the probability of No growth group (Supplementary Table 3).

### Antimicrobial Resistance

The proportions of bacteria from each group that were resistant to one, two, or > 2 of the tested antimicrobials are shown in Table 3. The NAS group included 66 isolates that were mostly susceptible (83% = 55/66) to all the antimicrobials tested (Table 3). Of the 23 *S. aureus* isolates, six (26%) were resistant to one antimicrobial. Resistance in the *Enterobacteriaceae* group was high: 64% of *E. coli* isolates were resistant to one antimicrobial; 18% were resistant to two antimicrobials; and 18% were resistant to > 2 antimicrobials. For the *Streptococcus* group, *S. uberis* was the most common (60% of isolates), and resistance to one and two antimicrobials was 39% and 33%, respectively. *Streptococcus agalactiae* (100% of isolates) were resistant to one antimicrobial. However, none of the *Streptococcus* spp. were resistant to > 2 antimicrobials.

**Table 3:** Distribution and prevalence of *Enterobacteriaceae* (*n* = 27), *Staphylococcus aureus* (*n* = 23), *Streptococcus* spp. (*n* = 30), non-aureus staphylococci (*n* = 66), *Bacillus* spp. (*n* = 27) Other (*n* = 21), and their antimicrobial resistance to one (R = 1), two (*R* = 2) and greater than two antimicrobial classes (R > 2).

**Bacterial group**	**Isolates (n)**	**Percentages**	**R = 1 AM^§^** **[n (%)]**	**R = 2 AM** **[n (%)]**	**R = >2 AM** **[n (%)]**
*Enterobacteriaceae*	27	100	14 (51)	3 (11)	13 (48)
*Escherichia coli*	11	40	7 (64)	2 (18)	2 (18)
*Enterobacter cloacae*	7	25	4 (57)	0 (0)	3 (43)
*Serratia marcescens*	5	18	0 (0)	0 (0)	5 (100)
*Enterobacter asburiae*	1	3	0 (0)	0 (0)	1 (100)
*Citrobacter species*	2	7	2 (100)	0 (0)	0 (0)
*Pantoea eucrina*	1	3	1 (100)	0 (0)	0 (0)
*Staphylococcus aureus*	23	100	6 (26)	0 (0)	0 (0)
*Streptococcus* spp.	30	100	14 (47)	7 (23)	0 (0)
*Streptococcus uberis*	18	60	7 (39)	6 (33)	0 (0)
*Streptococcus agalactiae*	7	23	7 (100)	0 (0)	0 (0)
*Streptococcus dysgalactiae*	1	3	0 (0)	0 (0)	0 (0)
*Streptococcus* species	4	13	0 (0)	1 (25)	0 (0)
Non-aureus staphylococci	66	100	10 (15)	1 (2)	0 (0)
*Staphylococcus chromogenes*	40	61	7 (18)	1 (3)	0 (0)
*Staphylococcus haemolyticus*	16	24	3 (19)	0 (0)	0 (0)
*Staphylococcus warneri*	2	3	0 (0)	0 (0)	0 (0)
*Staphylococcus sciuri*	2	3	0 (0)	0 (0)	0 (0)
*Staphylococcus simulans*	2	3	0 (0)	0 (0)	0 (0)
*Staphylococcus equorum*	1	2	0 (0)	0 (0)	0 (0)
*Staphylococcus hominis*	1	2	0 (0)	0 (0)	0 (0)
*Staphylococcus hyicus*	1	2	0 (0)	0 (0)	0 (0)
*Staphylococcus xylosus*	1	2	0 (0)	0 (0)	0 (0)
*Bacillus* spp.	27	100	10 (37)	1 (4)	1 (4)
*Bacillus megaterium*	10	20	8 (80)	0 (0)	0 (0)
*Bacillus pumilus*	6	12	0 (0)	0 (0)	0 (0)
*Bacillus cereus*	2	4	0 (0)	1 (50)	1 (50)
*Bacillus licheniformis*	1	2	1 (100)	0 (0)	0 (0)
*Bacillus subtilis*	1	2	0 (0)	0 (0)	0 (0)
*Bacillus species*	7	14	1 (14)	0 (0)	2 (29)
Others	21	100	13 (62)	1 (5)	9 (43)
*Acinetobacter venetianus*	2	4	0 (0)	0 (0)	2 (100)
*Aerococcus species*	1	2	1 (100)	0 (0)	0 (0)
*Aerococcus viridans*	5	10	1 (20)	0 (0)	0 (0)
*Enterococcus faecium*	3	6	0 (0)	1 (33)	2 (67)
*Exiguobacterium* sp[3]	1	2	0 (0)	0 (0)	0 (0)
*Lactococcus garvieae*	1	2	0 (0)	0 (0)	1 (100)
*Pasteurella multocida*	2	4	0 (0)	0 (0)	0 (0)
*Pseudomonas aeruginosa*	1	2	0 (0)	0 (0)	1 (100)
*Pseudomonas species*	1	2	0 (0)	0 (0)	1 (100)
*Pseudomonas stutzeri*	1	2	0 (0)	0 (0)	1 (100)
*Rothia terrae*	3	6	1 (33)	0 (0)	0 (0)
Total	194	100	57 (29)	12 (6)	22 (11)

*Bacterial species were cultured from milk samples collected from 151 clinical mastitis quarters and 268 apparently healthy control quarters from 18 dairy herds located in Northern Queensland, Southeast Queensland, and Victoria between March and June 2019*.

§ *Antimicrobial; Corynebacterium auriscanis, C. bovis, C. falsenii, C. xerosis, and Corynebacterium species were excluded from this analysis (11 isolates in total)*.

Resistance proportions (expressed as percentages) to each antimicrobial by bacterial group are depicted in Table 4. Resistance to penicillin among NAS was 12%, and resistance to gentamycin and oxacillin among *Streptococcus* spp. was 50 and 30%, respectively. *Enterobacteriaceae* resistance percentages to amoxicillin-clavulanic acid (33%), cephalothin (33%), pen-novobiocin (100%), and ampicillin (30%) were high. Of note, 0% of *Streptococcus* spp. and 9% of *S. aureus* isolates were resistant to penicillin. Gram-negative isolates were all susceptible to chloramphenicol.

**Table 4:** Counts and percentages antimicrobial resistance in bacterial groups.

	**Bacterial group (N)**					
**Antimicrobial**	**Non-aureus staphylococci (66^*†*^)**	***Enterobacteriaceae* (27^*†*^)**	***Staphylococcus aureus* (23^*†*^)**	***Streptococcus* spp. (30^*†*^)**	***Bacillus* spp. (27^*†*^)**	**Others (21^*†*^)**
TE	2^‡^	7	0	0	0	14
SXT	0	7	0	3	0	14
ENR	0	0	0	3	0	5
GEN	0	0	0	50	0	0
AMC	0	33	0	3	11	14
KF	0	33	0	0	7	24
NOV	3	100	0	0	0	24
CRO	0	0	0	0	11	5
PEN	12	NA	9	0	52	29
ERY	2	NA	17	3	0	7
OXA	0	NA	0	30	15	36
AMP	NA	30	NA	NA	NA	57
NEO	NA	4	NA	NA	NA	0
CHL	NA	0	NA	NA	NA	0

*Bacterial species were cultured from milk samples collected from 151 clinical mastitis quarters and 268 apparently healthy control quarters from 18 dairy herds located in Northern Queensland, Southeast Queensland, and Victoria between March and June 2019*.

† *Count*.

‡ *Percentages*.

*AMC, amoxicillin-clavulanic acid; AMP, ampicillin; CRO, ceftiofur; CHL, chloramphenicol; ENR, enrofloxacin; ERY, erythromycin; GEN, gentamicin; KF, cephalothin; NEO, neomycin; NOV, pen-novobiocin; OXA, oxacillin; PEN, penicillin; SXT, cotrimoxazole; TE, tetracycline; NA, Not preformed*.

**Figure 4:**
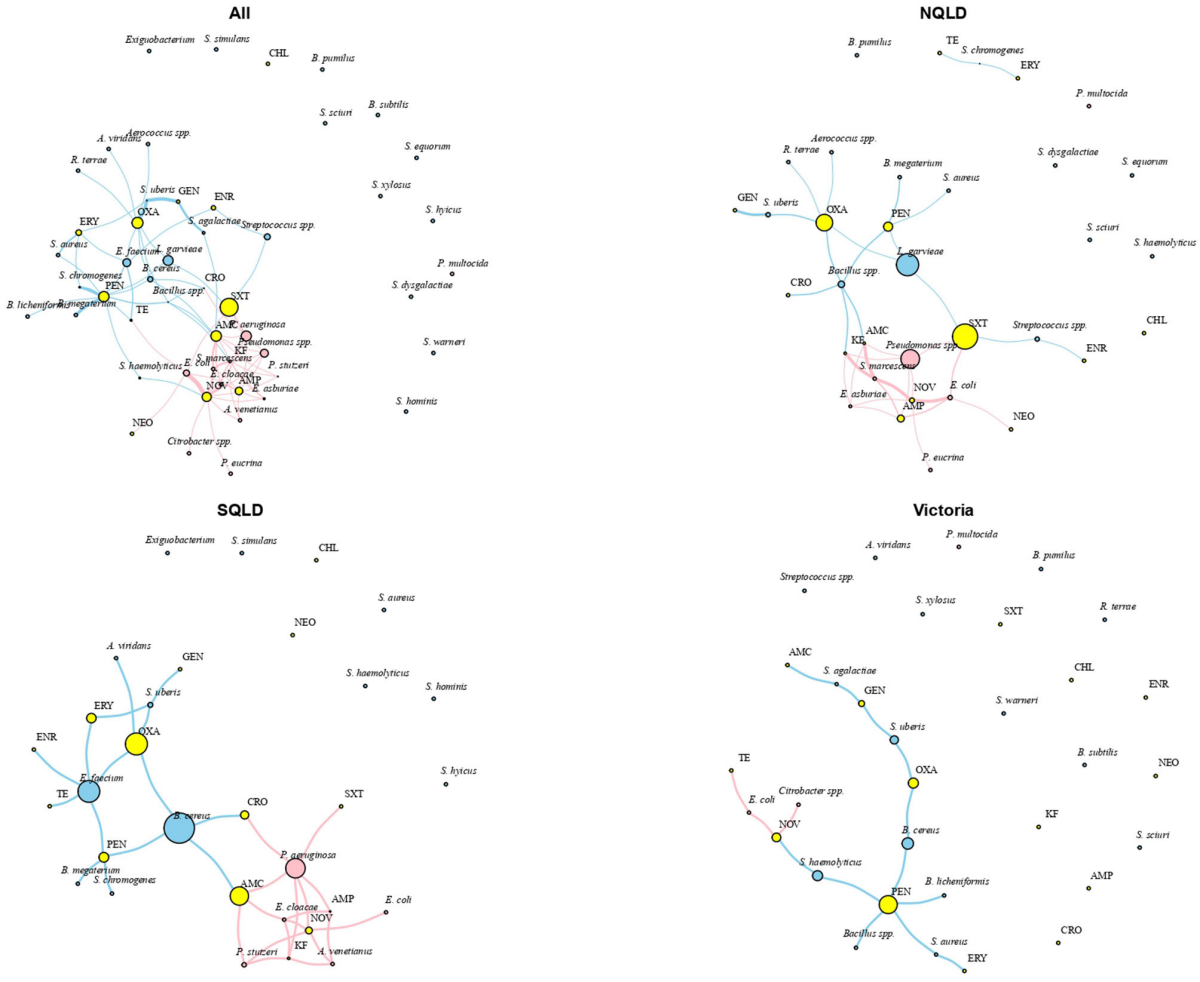
Social network analysis of antimicrobial resistance patterns of isolate groups and antimicrobial agents for the study population. Network constructed using multidimensional scaling and shortest path as the distance matrix with the size of the nodes proportional to antimicrobial agent and isolate group eigenvector values. The eigenvector score is a measure of isolate or antimicrobial agent importance based on their overall centrality degree in the network. Key: blue, Gram-positive bacteria; pink, Gram-negative bacteria; AMC, amoxicillin-clavulanic acid; AMP, ampicillin; CHL, chloramphenicol; CRO, ceftiofur; ENR, enrofloxacin; ERY, erythromycin; GEN, gentamicin; KF, cephalothin; NEO, neomycin; NOV, pen-novobiocin; OXA, oxacillin; PEN, penicillin; SXT, cotrimoxazole; TE, tetracycline. NQLD, Northern Queensland; SQLD, Southeast Queensland.

Irrespective of bacterial species type, various regional MDR patterns were observed. The most common MDR included neomycin, pen-novobiocin, or ampicillin. The resistance pattern in NQLD was centered on ampicillin and co-occurred with cotrimoxazole, neomycin, pen-novobiocin, amoxicillin-clavulanic acid, and cephalothin. For SQLD, resistance patterns were centered on pen-novobiocin and co-occurred with erythromycin and other around amoxicillin-clavulanic acid. No MDR pattern was observed for isolates from Victoria (Figure 4).

## Discussion

The major challenge facing the modern dairy industry is the pressure of minimizing the incidence of bovine mastitis. Investigations of mastitis etiology may help provide information about the bacteria present and inform possible approaches to control the disease. In the current study, the prevalence and probabilities of isolating bacteria of different types, and their association with animal (quarter) status, BMTSCC, and different regions were assessed. To our knowledge, this is the first Australian study to undertake a comparative analysis of dairy herds from regions in different climate zones in Australia. Previous studies were focused on collecting milk samples from herds in a defined location (14–16). Antimicrobial susceptibility of the mastitis bacteria was also evaluated.

Overall, microbiological results indicated that environmental mastitis bacteria were more prevalent than contagious bacteria (*S. aureus, S. agalactiae, C. bovis)*. However, contagious mastitis bacteria were more common in dairy farms in Victoria compared to NQLD and SQLD. The most commonly isolated bacteria from bovine milk samples were staphylococci, followed by streptococci spp., and *Enterobacteriaceae*. This is in agreement with other studies indicating that *S. aureus* and NAS are the most common cultured bacteria from bovine milk samples (32–34). NAS bacteria represented 32% of all isolated bacteria in the present study, which is similar to the findings of other international mastitis studies that reported predominance of NAS in dairy farms (35–37). Bovine mastitis caused by NAS usually remain as subclinical or mild clinical mastitis, but they can also cause severe local and systematic clinical signs (38). They are opportunistic pathogens found in the dairy farm environment and are part of the teat skin microbiota (38). However, NAS can cause persistent infections, which may result in increased SCC and decreased milk production (38).

Nine NAS species were identified in the present study, among which *S. chromogenes* was the most common, followed by *S. haemolyticus*. Previous studies on NAS from bovine milk have shown a diverse collection of NAS species. In recent studies, the frequently isolated NAS species from bovine milk were *S. chromogenes, S. haemolyticus, S. simulans, S. epidermidis*, and *S. xylosus* (39, 40). In this study, *S. chromogenes* was more frequently isolated than other NAS species from mastitic milk samples, indicating that this bacterium is the predominant NAS species causing clinical mastitis in the study population.

The current study showed that the herd's average BMTSCC influenced the probability of culturing NAS. NAS species were mainly isolated from herds with BMTSCC ≤150,000 cells/mL. These results are consistent with the literature and could be due to the lower risk of mastitis attributed to other (major) mastitis-associated bacteria in the herd (41). In a large field survey of milk sample culture, collected between 1992 and 2007 from dairy herds in New York, United States, Schukken et al. (41) reported that in herds with BMTSCC <200,000 cells/mL, ~18% of cells in bulk milk were shed from NAS culture-positive animals. The authors also reported that a high proportion of NAS infections in the herd could result in a moderate or high BMTSCC. Similar results were also reported by Dufour et al. (42). Contrarily, 22% of NAS isolates cultured in the current study were from control samples; most of them from apparently healthy control cows. NAS species have previously been isolated from apparently healthy quarters. In the Netherlands (43) and Germany (35), NAS were isolated from 10% and 11% of apparently healthy quarters, respectively. Although isolating NAS from apparently healthy quarters may support the beneficial role of these bacteria in udder health, conflicting evidence regarding the virulence potential of these bacteria still raises controversy regarding this hypothesis (44). In Argentina, Isaac et al. (45) reported that some NAS species isolated from healthy quarters have the ability to inhibit biofilm formation of mastitis-associated bacteria, but do not have direct antimicrobial effects against major mastitis pathogens, *in vitro*. Because the NAS species isolated from apparently healthy quarters were common in the current study and in other bovine mastitis studies, further research is needed to evaluate these species as commensal healthy milk microbiota.

This study also revealed *Streptococcus* spp. were common in clinical cases of mastitis, which is in agreement with studies from North America (46, 47). Among the streptococcal species isolated in the present study, *S. uberis* was the most frequent, representing 11% of the total bacteria. This is in line with a study in south eastern Australia that showed *S. uberis* to be the most common bacterial species cultured in bovine mastitis cases (14). Further, *S. uberis* was the most frequent Gram-positive bacterial species identified in clinical mastitis cases in New South Wales (15). Similar results have been reported internationally (35, 48). These observations suggest that *S. uberis* continues to be extensively responsible for mastitis in Australian dairy herds. The major challenge with *S. uberis*-induced bovine mastitis is the variety of transmission routes of this isolate. This microorganism has been classified as an environmental mastitis pathogen, yet it commonly manifests itself in both environmental and contagious forms (49). A possible contagion route is via contaminated milking machines (49). Also, *S. uberis* has the ability to survive in diverse environments such as pasture, bedding, fecal material, and various body sites of the cow (50). The prevalence of *S. uberis* is high in pasture-based dairying systems in countries such as New Zealand (51, 52) and during pasture season in the Netherland (53). Bovine feces are a major source of *S. uberis* (54), and cows in pasture may maintain a contamination cycle of *S. uberis* through the feces. The Australian dairy industry is mainly pasture-based, which may explain the high prevalence of *S. uberis* in the present study and one other recent study (14). In contrast, the Portuguese dairy industry is largely barn-based, rather than pasture, and this may explain the lower rates of *S. uberis* infections in Portugal's dairy industry (55).

It was notable in this study that most of the *S. agalactiae* isolates were cultured from milk collected from mastitis cases in two Victorian dairy herds. *Streptococcus agalactiae* was not detected in any milk samples collected from dairy farms in Queensland. This finding is in accordance with a recent study conducted in dairy herds in Queensland (16). The prevalence of *S. agalactiae* in Queensland has decreased over the last four decades, as it was previously reported in a high proportion of dairy farms and was the main cause of mastitis in many dairy herds in the region (56, 57). *Streptococcus aureus* was detected in 11% of the milk samples in the present study, more frequently in Victoria than in Queensland dairy farms. The results here suggest that the incidence of contagious mastitis due to *S. agalactiae* and *S. aureus* should be monitored in Victorian dairy farms for effective mastitis control.

The *Enterobacteriaceae* group were mainly isolated from mastitis cases in this study. This group has been found to be a very common cause of clinical mastitis in dairy cows (32). Among the *Enterobacteriaceae* group the percentage of *E. coli* positive samples in mastitis cases was significantly higher compared to other Gram-negative bacteria. Other research has found that *E. coli* is the most common coliform bacteria in clinical mastitis, accounting for 80% of the coliform mastitis cases (48). The probability of culturing *Enterobacteriaceae* in our study tended to be higher in herds with low BMTSCC (≤150,000 and 150–300,000 cells/mL) than in herds with high BMTSCC (>300,000 cells/mL). A similar finding was reported by Barkema et al. (58) where *E. coli* was the predominant microorganism that caused clinical mastitis in herds with low BMTSCC. Miltenburg et al. (59) also reported that *E. coli* mastitis incidence was higher in herds with a low BMTSCC (<150,000 cells/mL). This might be due to *E. coli* infections usually occurring in early lactation, when immunosuppression caused by metabolic deficiencies is more common than in later lactation (60, 61) or in extremely low SCC, which lower the host's resistance (62).

In the current study, 28% of mastitis milk samples yielded negative bacterial culture. PCR has the potential to detect mastitis pathogens from culture-negative milk samples (63). “No growth” cultures may occur due to the low concentration of bacteria in the milk samples (64), or the presence of anaerobic bacteria. Neither herd's region nor BMTSCC influenced the probability of culture-negative milk samples in the present study. Thus, mastitis milk samples may be culture-negative for the different reasons mentioned above.

NAS were more prevalent in Queensland dairy herds compared to Victoria, unlike *S. aureus*. The effectiveness of the mastitis control programs could explain the differences in prevalence between regions. The relative importance of NAS increases when the prevalence of major mastitis pathogens decreases (4). In Finland, as *S. aureus* IMI have decreased in mastitis control programs, prevalence of NAS has increased (37). NAS are part of the teat skin microbiota and colonize the teat apex (44). Therefore, habitation of the teat apex by NAS may inhibit the growth of mastitis-associated *S. aureus*.

In this study, *S. aureus* isolates showed high susceptibility to the antimicrobials tested, with only two penicillin-resistant *S. aureus* isolates, and non-resistant to two or more antimicrobials. This finding is consistent with a recent study in Victoria (65). Similarly, NAS species showed high antimicrobial susceptibility, with only 2% of the isolates resistant to two or more antimicrobials; however, 12% were resistant to penicillin, which is relatively high compared to other bacterial species. This is in agreement with other studies evaluating antimicrobial susceptibility of bovine mastitis bacteria, where NAS species were reported to have high resistance rates to penicillin (1, 66).

All *Streptococcus* spp. in this study were found to be susceptible to penicillin. This finding is consistent with one other previous dairy herd survey (1). Penicillin is the antimicrobial of choice in the treatment of bovine mastitis caused by *Streptococcus* spp. (10, 67, 68). Therefore, it can be recommended from the current results that penicillin can still be used as a front-line drug to treat streptococcal mastitis in dairy herds in Australia. However, *Streptococcus* spp. showed a high resistance rate to oxacillin, a semi-synthetic beta lactam belonging to the group of isoxazolyl penicillins (69). Most Gram-positive bacteria are reportedly inhibited at low oxacillin concentration (69). Both NAS and *S. aureus* in the current study were susceptible to oxacillin, but 30% of *Streptococcus* spp. were resistant.

In this study, *Enterobacteriaceae* species showed high resistance rates (48%) to two or more antimicrobials. Further, 30% of *Enterobacteriaceae* were resistant to ampicillin, and 33% to amoxicillin-clavulanic acid. High resistance profiles to beta lactam antibiotics have been reported in many other studies of bovine mastitis *Enterobacteriaceae* species (32, 70). On the other hand, all the *Enterobacteriaceae* species were susceptible to ceftiofur. Therefore, this antimicrobial can be used to treat severe clinical bovine mastitis caused by *Enterobacteriaceae* bacteria, particularly *E. coli*, in Australian dairy herds.

Gentamicin, enrofloxacin, and chloramphenicol were tested in this study for public health concerns. In Australia, these antimicrobials are regulated and their use is restricted to human consumption (71). Half the *Streptococcus* spp. were resistant to gentamicin and 3% were resistant to enrofloxacin, while no Gram-negative bacteria were resistant to chloramphenicol. This finding indicates that resistance to gentamicin remains high in Australia.

Antimicrobials are used to treat and prevent bovine mastitis in Australia (72). However, little effort has been made to monitor the emergence of AMR in mastitis bacteria, with few local studies only having investigated the prevalence of AMR for *S. aureus* (65) and NAS (73). This lack of data prevents conclusions from being drawn about the changes in local resistance patterns over time. Our findings indicate possible regional differences in the phenotypes of MDR patterns in the study isolates. Of interest is to investigate whether similar genotypic differences exist both at the isolate and regional levels. The availability of such information would allow a better understanding of MDR resistance on dairy farms.

This study has limitations that should be mentioned. First, the use of the CMT test to confirm healthy quarters may give rise to false negative and false positive results, as previously reported; however, the test performed well in terms of negative predictive value (20). This may explain the isolation of some potential mastitis pathogens from control milk samples. NAS often cause slight increases in quarter milk SCC (33), which may result in false negatives in the CMT test. The results described here can be extrapolated to herds that are similar to the study population. Extrapolation and generalizing the study results to other herds requires a larger study and enrolment of more herds with greater numbers of milk samples collected from cows. Selection bias is likely to have been introduced due to the convenience selection of the study herds. Although it could be argued that the study herds are typical Australian dairy herds, the effect of selection bias is likely that the effect is bidirectional. If the study herds had poor (or effective) mastitis management strategies, then the results from this study would have overestimated (or underestimated) the prevalence and strength of association between bacterial groups with regions in BMTSCC. Finally, the herds zones may not be representative of the entire Australian dairy industry, as the herds sampled in the study were based on being able to build collaborative interactions with the farmers, rather than on even industry representation. Nevertheless, herd type, breed of dairy cow, and herd size were representative of the Australian herds.

## Conclusion

The present study recorded high overall prevalence of environmental bacteria in bovine milk samples collected in different regions in Australia. The findings provide an opportunity to study the prevalence of bacterial species collected from dairy herds in Australia. Overall, staphylococci spp. were the most frequent bacteria, followed by streptococci spp., and *Enterobacteriaceae*. Contagious mastitis bacteria, including *S. agalactiae* and *S. aureus*, were more prevalent in Victorian compared to Queensland dairy herds. Although antimicrobial susceptibility tests showed overall low resistance profiles, particular attention should be paid to *Enterobacteriaceae*, which showed high resistance rates to various antimicrobials used in this study, notably ampicillin and amoxicillin-clavulanic acid. There is a lack of AMR data recorded for bacterial species in Australian dairy cattle; therefore, further effort is warranted to monitor AMR patterns for effective AMR control programs.

## Data Availability Statement

The original contributions presented in the study are included in the article/Supplementary Material, further inquiries can be directed to the corresponding author/s.

## Ethics Statement

The animal study was reviewed and approved by the University of Queensland Animal Ethics and National Guidelines. A verbal informed consent was obtained from the owners for the participation of their animals in this study.

## Author Contributions

HA-h: study design, sample collection, sample processing, and data analyses. JA: study design, sample collection, and data analyses. SR: data analysis and interpretation. RM: assisted with data interpretation. All authors contributed to drafting of the manuscript.

## Funding

This study was funded by Science with Impact Funding, University of Queensland, the Australian Subtropical Dairy Board, Terragen Biotech Ltd. Pty., and the Saudi Government—University of Jeddah.

## Conflict of Interest

This study received funding from Terragen Biotech Pty. Ltd. The funder was not involved in the study design, collection, analysis, interpretation of data, the writing of this article or the decision to submit it for publication. The authors declare that the research was conducted in the absence of any commercial or financial relationships that could be construed as a potential conflict of interest.

## Publisher's Note

All claims expressed in this article are solely those of the authors and do not necessarily represent those of their affiliated organizations, or those of the publisher, the editors and the reviewers. Any product that may be evaluated in this article, or claim that may be made by its manufacturer, is not guaranteed or endorsed by the publisher.
