# Supplementary material for: Bacteria Isolated From Milk of Dairy Cows With and Without Clinical Mastitis in Different Regions of Australia and Their AMR Profiles
Source: Front Vet Sci. 2021 Nov 4;8:743725. doi: 10.3389/fvets.2021.743725 (PMC8600363; doi:10.3389/fvets.2021.743725)
Supplement: Supplementary file 1 [file Table_1.docx]

# Supplementary Tables

Supplementary Table 1: Counts of non-*aureus* staphylococci isolates (n= 66) cultured from mastitis quarters, external control, and internal control. Milk samples were obtained from dairy cattle originating from 18 dairy herds in Northern Queensland, Southeast Queensland and Victoria between March and June 2019.

| Non-*aureus* staphylococci | Mastitis | External control | Internal control | Total |
| --- | --- | --- | --- | --- |
| *Staphylococcus chromogenes* | 15 | 17 | 8 | 40 |
| *Staphylococcus haemolyticus* | 1 | 10 | 5 | 16 |
| *Staphylococcus warneri* | 2 | 0 | 0 | 2 |
| *Staphylococcus sciuri* | 2 | 0 | 0 | 2 |
| *Staphylococcus simulans* | 0 | 0 | 2 | 2 |
| *Staphylococcus equorum* | 0 | 0 | 1 | 1 |
| *Staphylococcus hominis* | 1 | 0 | 0 | 1 |
| *Staphylococcus hyicus* | 0 | 0 | 1 | 1 |
| *Staphylococcus xylosus* | 0 | 0 | 1 | 1 |
| Total | **21** | **27** | **18** | **66** |

Supplementary Table 2. Results from conditional multilevel logistic model (including 95% Confidence interval (CI) in parentheses) showing variables that influence the odds of culturing a bacterial group from mastitis and healthy quarters, between herd’s region, and average bulk milk somatic cell counts (BMTSCC). Bacterial species were cultured from milk samples collected from 151 clinical mastitis quarters and 268 apparently healthy control quarters from 18 dairy herds located in Northern Queensland, Southeast Queensland, and Victoria between March and June 2019.

| Variable | *Enterobacteriaceae* | *Staphylococcus aureus* | *Streptococcus* spp. | Non-*aureus* staphylococci | *Bacillus* spp. | No growth |
| --- | --- | --- | --- | --- | --- | --- |
| Sample type | | | | | | |
| Control | reference | reference | reference | reference | reference | reference |
| Case | 6.7 (2.70, 16.72) ** | 2.08 (0.59, 7.34) ^ns^ | 9.37 (3.54, 24.85) ** | 0.49 (0.24, 0.99) * | 3.62 (1.05, 12.45) ^ns^ | 0.43 (0.29, 0.64) ** |
| Herds regions | | | | | | |
| Northern Queensland | reference | reference | reference | reference | reference | reference |
| Southeast Queensland | 0.60 (0.24, 1.52) ^ns^ | 1.85 (0.14, 24.38) ^ns^ | 1.28 (0.35, 4.66) ^ns^ | 0.63 (0.22, 1.86) ^ns^ | 1.35 (0.04, 42.69) ^ns^ | 1.04 (0.56, 1.92) ^ns^ |
| Victoria | 0.51 (0.20, 1.32) ^ns^ | 6.6 (1.0, 57.08) * | 1.22 (0.38, 3.93) ^ns^ | 0.15 (0.04, 0.5) ** | 3.37 (0.3, 37.33) ^ns^ | 1.17 (0.67, 2.05) ^ns^ |
| Herd BMTSCC | | | | | | |
| <150,000 cells/mL | reference | reference | reference | reference | reference | reference |
| 150,000-300,000 cells/mL | 1.20 (0.52, 2.76) ^ns^ | 4.70 (0.47, 46.91) ^ns^ | 12.60 (1.50, 106.18) ** | 0.25 (0.09, 0.69) ** | 2.09 (0.12, 34.95) ^ns^ | 0.94 (0.46, 1.95) ^ns^ |
| >300,000 cells/mL | 0.21 (0.02, 1.72) ^ns^ | 3.17 (0.16, 62.18) ^ns^ | 30.51(3.17, 293.70) * | 0.16 (0.04, 0.61) ** | 11.46 (0.43, 308.72) ^ns^ | 0.64 (0.38, 1.08) ^ns^ |

ns= not significant * P<0.05; ** P<0.001; spp. Species.

Supplementary Table 3. Results from conditional multilevel logistic model (including 95% Confidence interval (CI) in parentheses) showing variables that influence the probability (expressed as percentages) of culturing a bacterial group from mastitis and healthy quarters, between herd’s region, and average bulk milk somatic cell counts (BMTSCC). Bacterial species were cultured from milk samples collected from 151 clinical mastitis quarters and 268 apparently healthy control quarters from 18 dairy herds located in Northern Queensland, Southeast Queensland, and Victoria between March and June 2019.

| Variable | *Enterobacteriaceae* | *Staphylococcus aureus* | *Streptococcus* spp. | Non-*aureus* staphylococci | *Bacillus* spp. | No growth |
| --- | --- | --- | --- | --- | --- | --- |
| Sample type | | | | | | |
| Control | reference | reference | reference | reference | reference | reference |
| Case | 87% (73%, 94%) | 67% (37%, 88%) | 90% (78%, 96%) | 33% (19%, 50%) | 78% (51%, 93%) | 30% (22%, 39%) |
| Herds regions | | | | | | |
| Northern Queensland | reference | reference | reference | reference | reference | reference |
| Southeast Queensland | 38% (19%, 60%) | 65% (12%, 96%) | 56% (26%, 82%) | 39% (18%, 65%) | 57% (4%, 98%) | 51% (36%, 66%) |
| Victoria | 33% (16%, 56%) | 87% (43%, 98%) | 55% (27%, 80%) | 13% (4%, 34%) | 77% (23%, 97%) | 54% (40%, 67%) |
| Herd BMTSCC | | | | | | |
| <150,000 cells/mL | reference | reference | reference | reference | reference | reference |
| 150,000-300,000 cells/mL | 54% (34%, 73%) | 76% (14%, 98%) | 93% (60%, 99%) | 20% (8%, 41%) | 92% (30%, 100%) | 49% (31%, 66%) |
| >300,000 cells/mL | 17% (2%, 63%) | 82% (32%, 98%) | 97% (76%, 100%) | 14% (4%, 38%) | 68% (11%, 97%) | 39% (27%, 52%) |

spp. Species.
